# High-Precision Propagation-Loss Measurement of Single-Mode Optical Waveguides on Lithium Niobate on Insulator

**DOI:** 10.3390/mi10090612

**Published:** 2019-09-15

**Authors:** Jintian Lin, Junxia Zhou, Rongbo Wu, Min Wang, Zhiwei Fang, Wei Chu, Jianhao Zhang, Lingling Qiao, Ya Cheng

**Affiliations:** 1 State Key Laboratory of High Field Laser Physics, Shanghai Institute of Optics and Fine Mechanics, Chinese Academy of Sciences, Shanghai 201800, China; jintianlin@siom.ac.cn (J.L.); rbwu@siom.ac.cn (R.W.); chuwei@siom.ac.cn (W.C.); jhzhang@siom.ac.cn (J.Z.); qiaolingling@siom.ac.cn (L.Q.); 2 State Key Laboratory of Precision Spectroscopy, East China Normal University, Shanghai 200062, China; 5218092020026@stu.ecnu.edu.cn (J.Z.); mwang@phy.ecnu.edu.cn (M.W.); zwfang@phy.ecnu.edu.cn (Z.F.); 3 XXL—The Extreme Optoelectromechanics Laboratory, School of Physics and Materials Science, East China Normal University, Shanghai 200241, China; 4 School of Optoelectronics, University of Chinese Academy of Sciences, Beijing 100049, China; 5 Collaborative Innovation Center of Extreme Optics, Shanxi University, Taiyuan 030006, Shanxi, China

**Keywords:** lithium niobate, waveguide, photonic integrated circuit, optical lithography, chemomechanical polish

## Abstract

We demonstrate the fabrication of single-mode optical waveguides on lithium niobate on an insulator (LNOI) by optical patterning combined with chemomechanical polishing. The fabricated LNOI waveguides had a nearly symmetric mode profile of ~2.5 µm mode field size (full-width at half-maximum). We developed a high-precision measurement approach by which single-mode waveguides were characterized to have propagation loss of ~0.042 dB/cm.

## 1. Introduction

Pursuing a photonic integrated circuit (PIC) has been taking place in the past few decades. inspired by the enormous success of electronic integration [1,2]. One of the key building blocks for realizing PICs is single-mode optical waveguides, by which the transportation and manipulation of photons can be realized in compact and stable optical networks. Major requirements for waveguides are low optical loss, and high tunability and nonlinearity. As an important platform for PICs, silicon-based optical waveguides provide high field confinement [3]. However, lack of second-order nonlinearity and electro-optic coefficients, and relatively high propagation loss are obstacles in silicon photonic applications. To this end, crystalline lithium niobate is almost an ideal candidate due to its broad transparent window, and high electro-optic and nonlinear coefficients [4,5].

Early investigations in establishing PICs on lithium niobate (LN) focused on titanium-diffused LN waveguides with only subtle refractive index contrast, of which the large bending radius posed a scalability challenge [4]. On the other hand, several attempts were made on fabricating high-quality nanophotonic structures on lithium niobate on an insulator (LNOI) with either Ar^+^ ion etching or diamond dicing. Results were not promising due to high surface roughness on the sidewalls of the fabricated structures [6,7]. The problem was tackled by employing focused-ion-beam (FIB) writing to realize a surface roughness of a few nanometers on the sidewalls of nanophotonic structures, resulting in LN microresonators with quality (Q) factors on the order of 10^6^ [8,9,10,11,12]. Afterwards, combinations of either ultraviolet lithography or electron-beam lithography with reactive ion etching produced similar results on LNOI [12,13,14,15,16]. Nevertheless, nanometer-scale roughness left behind by dry ion etching was still an obstacle to further reduce the optical loss of the LNOI waveguide to below 10^−1^ dB/cm. In comparison with the intrinsic absorption loss of LN crystals, which is on the order of 10^−3^ dB/cm, the current optical loss could be improved by two orders of magnitude if scattering loss on the surface could be substantially eliminated. This provides a strong incentive to further suppress surface roughness on the sidewalls [17].

We recently developed a fabrication approach that allows to fabricate both high-Q factor LN microdisks and low-loss LN ridge waveguides [18,19,20]. The technique begins with femtosecond laser micromachining for patterning a hard chromium (Cr) mask coated on the LNOI, followed by chemomechanically polishing (CMP) the LNOI sample to transfer the generated pattern onto the LNOI. The fabricated waveguide in this manner showed surface roughness of Rq~0.452 nm and propagation loss of 0.027 dB/cm [19,20]. Unfortunately, LN waveguides do not support single-mode propagation due to its relatively large cross-sectional dimensions, which is typically on the submicrometer scale but not ~100 nm scale. Here, we converted the multimode LN waveguides to single-mode waveguides by covering them with a cladding layer, and examined the mode profiles and propagation losses in single-mode waveguides.

## 2. Fabrication

**Figure 1:**
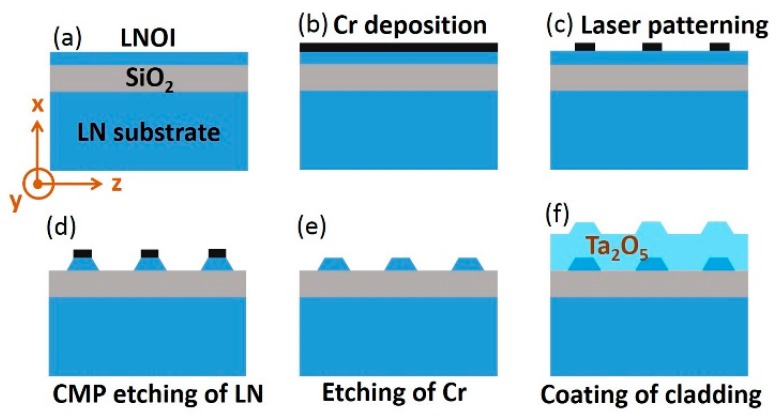
Process flow of lithium niobate on insulator (LNOI) waveguide fabrication. (**a**) Structure of LNOI substrate; (**b**) Depositing a layer of Cr film on LNOI; (**c**) Patterning the Cr film by femtosecond laser ablation for producing the mask of waveguides; (**d**) Transferring the mask pattern to LNOI by chemo-mechanical polish; (**e**) Removing the remaining Cr film by wet chemical etching; (**f**) Coating the waveguides with a layer of Ta_2_O_5_ to form the cladding.

A commercially available X-cut LNOI wafer was used in the fabrication of the ridge waveguides. The LNOI wafer was produced by crystal ion slicing, and then bonded onto a silica layer of 2 μm thickness supported by a bulk LN substrate of 500 μm thickness. The structure of the LNOI wafer is shown in Figure 1a. The ridge waveguide was oriented along the Y axis. In such a configuration, the transverse-electric (TE) mode in the waveguide experiences the extraordinary refractive index of the LN crystal. The process flow for fabricating the ridge waveguides has five steps, as illustrated in Figure 1.

**Figure 2:**
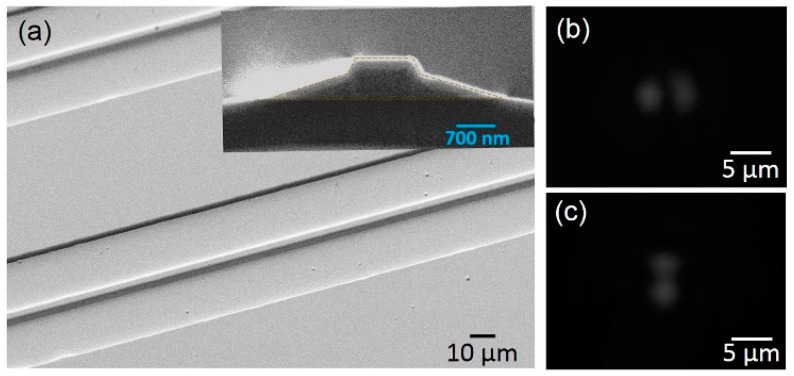
(**a**) Scanning-electron-microscope (SEM) images of lithium niobate (LN) waveguide before coating the Ta_2_O_5_ cladding layer) showing very smooth sidewalls. Inset: waveguide cross-section. Spatial distributions of (**b**) transverse-electric (TE) and (**c**) transverse-magnetic (TM) modes.

First, a wear-resisting Cr layer with a thickness of 600 nm was coated onto the LNOI wafer by magnetron sputtering, as shown in Figure 1b. Note that Cr hardness was much higher than that of LN, making Cr good hard mask material for protecting the LN underneath in the CMP process. Second, the Cr layer was patterned into stripes with a width of ~1 µm by femtosecond laser ablation, as shown in Figure 1c. Femtosecond laser ablation was performed at a repetition rate of 250 kHz and an average power of 0.05 mW. An objective lens (Model: M Plan Apo NIR, Mitutoyo Corporation, Kanagawa, Japan) with a numerical aperture (NA) of 0.7 was used to focus the laser pulses, creating a focal spot of 1 µm diameter on the sample. This objective lens was mounted onto a one-dimensional (1D) motion stage (Model: ANT130-110-L-ZS, Aerotech Inc., Hanover, MD, USA), which traveled in the vertical (Z) direction at a resolution of 100 nm to ensure accurate focusing onto the sample surface. The sample was mounted onto an XY stage (Model: ABL15020WB and ABL15020, Aerotech Inc.) with a translation resolution of 100 nm. The Cr was patterned by scanning the focal spot across the areas according the designed patterns. All motion stages were computer-programmable. The laser power we chose was sufficient for ablating Cr, but insufficient for ablating the LN crystal, because the damage threshold of LN was higher than that of Cr [21]. This characteristic ensured that, in the ablation of Cr, the LN thin film stayed intact. Afterwards, the LNOI wafer was subjected to CMP, which was carried out using a wafer-polishing machine. An LN ridge structure was obtained after CMP, as shown in Figure 1d. A smooth sidewall with an average roughness of ~0.5 nm was attainable by CMP. The Cr mask was removed via chemical etching by immersing the sample into a Cr etching solution for 4 min, as shown in Figure 1e. Lastly, a layer of Ta_2_O_5_ was coated onto the sample to create a suitable refractive index contrast for ensuring single-mode waveguiding, as shown in Figure 1f. The scanning-electrical-microscope (SEM) image of the fabricated LN ridge waveguide is presented in Figure 2a, and the waveguide cross-section is shown in the inset. Although the ablated resolution of Cr by femtosecond laser was on the magnitude of several tens of nanometers, the edge of the Cr stripes also endured CMP polishing. Therefore, this roughness did not transfer onto the LN ridge sidewall. The top width of the waveguide was determined to be ~1.0 µm, whereas the bottom width was measured as ~4.2 µm.

## 3. Characterization

Spatial modes were excited and characterized by coupling the waveguide with a 1550 nm wavelength laser using a fiber lens. The polarization state of the input light was controlled by an in-line polarization controller. The light transmitted from the output port of the waveguide was collected by an objective lens with NA = 0.3. A beam expander was introduced behind the objective lens, and it projected the image of the output port onto an infrared charge-coupled device (CCD) (InGaAs camera, HAMAMATSU Inc., Shizuoka, Japan). The spatial distributions of transverse-electric (TE) and transverse-magnetic (TM) modes were captured by the CCD, as shown in Figure 2b,c, respectively. The waveguide supported high-order spatial modes for the TE and TM modes owing to the high refractive index of LN and the large transverse dimensions of the waveguide.

**Figure 3:**
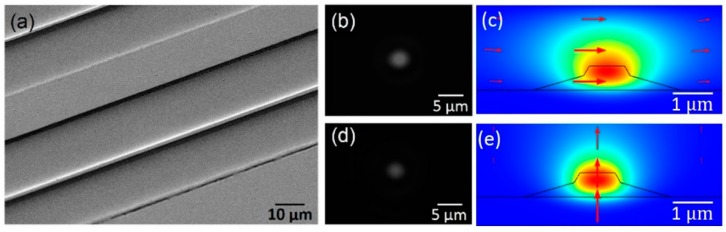
(**a**) SEM image of waveguide covered with Ta_2_O_5_. (**b**) Measured and (**c**) calculated TE mode profiles. (**d**) Measured and (**e**) calculated TM mode profiles.

To produce single-mode LNOI waveguides for both the TE and the TM mode, Ta_2_O_5_ (refractive index 2.057) of ultralow loss [22] was deposited onto the fabricated sample by electron-beam evaporation. Figure 3a shows the scanning electron microscope (SEM) image of the LN ridge waveguide covered with the Ta_2_O_5_ cladding layer of 3.5 µm thickness. A single-mode spatial profile was obtained for TE mode, as shown in Figure 3b, which was consistent with the calculated mode profile in Figure 3c. Similarly, such a waveguide supported single-mode propagation for TM mode as well, as shown in Figure 3d and evidenced by the corresponding calculation result in Figure 3e. The full width at half maximum (FWHM) of the TE mode was measured to be ~2.5 µm, and the FWHM of the TM mode was ~2.3 µm.

Traditionally, the propagation loss of a ridge waveguide can be measured based on the Fabry–Perot (FP) cavity-measurement or direct cut-back method [7,23,24,25]. However, ultralow loss of our chemomechanically polished LNOI waveguide could not be determined by the above methods due to insufficient precision. To overcome this difficulty, we developed a unique technique that allowed us to reliably measure nearly inappreciable loss in the on-chip LNOI waveguide.

**Figure 4:**
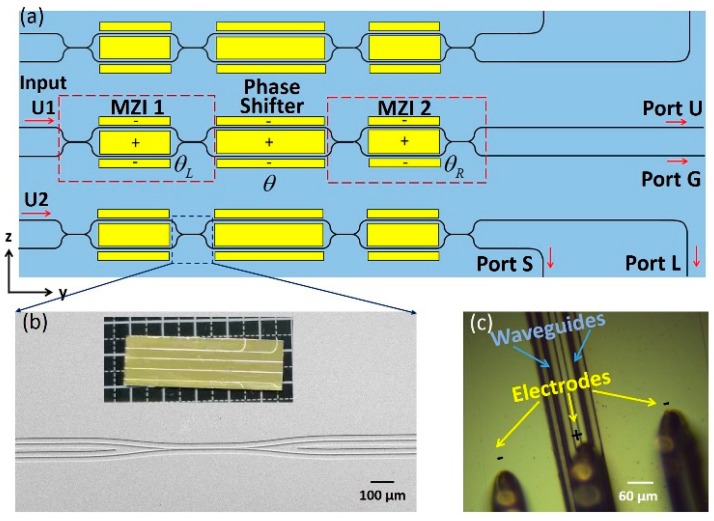
(**a**) Device layout. Here, phase differences *θ*_L_, *θ*, and *θ*_R_ were contributed by fabrication imperfections of interferometer arms and of electro-optic (EO) modulation. (**b**) SEM image of directional coupler; inset: overview photograph of fabricated device consisting of three beam splitters. (**c**) Optical-microscope image of electrodes contacted by three pins.

Our high-precision loss-measurement method was established based on the design in Figure 4a. The device was composed of three beam splitters aligned in a vertical array, each of which consisting of two identical electro-optic (EO) Mach–Zehnder interferometers (MZIs) bridged by an EO phase shifter. A similar design was used to produce high-extinction-ratio beam splitters immune to fabrication imperfections [26,27]. The beam splitter was fabricated on an X-cut LNOI chip with its optic axis oriented perpendicularly to the MZI arms, as in Figure 4a. The beam-splitting ratio of the fabricated directional coupler (see Figure 4b) was designed to be 7:3. The output arms of different lengths were fabricated to differentiate propagation loss, i.e., one arm was 12 mm longer than the other. After fabrication of the LNOI waveguides, Au electrodes with a thickness of 200 nm were added by magnetron sputtering followed by space-selective patterning via femtosecond laser ablation, as shown in Figure 4c. Gap *d* between the Au electrodes in each MZI was set as 10 µm, symmetrically arranging the two electrodes on both sides of the LNOI waveguides. The lengths of the interferometer arms of MZI 1, Phase Shifter, MZI 2, which were sandwiched between the Au electrodes, were 2, 11, and 2 mm, respectively. A photograph of the fabricated device captured by a digital camera is shown in the inset of Figure 4b. The total length of the chip was ~ 30 mm.

**Figure 5:**
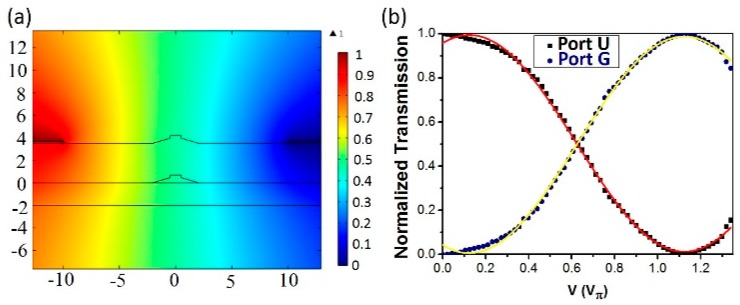
(**a**) Calculated normalized electric-field distribution in cross-sectional plane of LNOI waveguide when applying voltage on Au microelectrodes, showing that the electric field was almost parallel to the LN optic axis. (**b**) Normalized transmission spectra of output Ports U and G as a function of applied voltage V, featuring sinusoidal-like curve.

To characterize the EO response of the beam splitter, the telecom laser (New Focus Inc., San Jose, CA, USA, Model TLB 6728) with a pure TE mode at 1550 nm wavelength was coupled into input Port U1 of the beam splitter through a fiber lens. The output beam was first collimated by a 50× objective lens (Model: M Plan Apo NIR, Mitutoyo Corporation) of 0.42 NA, and then sent into an optical spectrum analyzer (OSA; YOKOGAWA Inc., Tamba City, Japan, Model AQ6370D, dynamic range 45 dB). With this arrangement, the electric field was parallel to the optic axis of the LN, as evidenced in Figure 5a. Phase difference *ϕ* between the waves exiting from the two interferometer arms could be expressed as:

(1) $$\phi =\frac{2\pi }{\lambda }n_{\mathrm{eff}}^{3}r_{33}\frac{V}{d}l$$

Here, *λ* is wavelength (i.e., 1550 nm), *n*_eff_ is the effective refractive index of 2.05, *r*_33_ (= 30.8 × 10^−12^ m/V) is the largest electro-optic coefficient of LN, and *V* is the applied voltage. The minimal tuning step of the direct current voltage was set to 0.02 V. Measured half-wave voltages *V*_π_ were 6.7 V for the phase shifter and 36.8 V for MZIs 1 and 2. The results agreed well with the numerical calculation. Next, we tuned the splitting ratio for MZIs 1 and 2 to a precise 50:50 by using the following procedure [26]:

1. Adjust voltage on phase shifter to minimize the output power of output Port G.
2. Scan voltages on MZIs 1 and 2 to minimize the output power of Port G.
3. Repeat Steps 1 and 2, if necessary, until the minimal power in output G is zero, and maximal power in Port U is as large as possible.

In our experiment, the extinction ratio between Ports G and U was determined to be ~40 dB based on the experiment curve in Figure 5b, which was obtained by varying phase difference *θ*, giving rise to an oscillating power curve expressed by [26]:

(2) $$P_{U}=\frac{1}{2}\left(1-\cos\theta \right)$$

The measured output power at Port U in Figure 5b nicely followed the cosine curve obtained using Equation (2), and that at Port G remained sinusoidal.

To measure the propagation loss of the single-mode waveguide, the laser was coupled to input Port U2 of the bottom beam splitter in Figure 4a, and both MZIs were tuned at a 50:50 splitting ratio via EO modulation. Then, power output from Port S was tuned to be maximal by adjusting the voltage applied on the phase shifter. Finally, power output from Port S was tuned to be half the maximal value. In other words, phase difference *θ* between the two arms of the phase shifter in the bottom beam splitter was π/2, and the powers injected into output Ports S and L were the same. Propagation loss was measured to be 0.042 ± 0.02 dB/cm by comparing the powers at output Ports S (averaged counts: 3943) and L (averaged counts: 3898). Median measured error was originated from the minimal tuning step of the voltage source.

## 4. Conclusions

To conclude, we fabricated single-mode LNOI waveguides with a propagation loss of ~0.042 dB/cm and a mode field size of ~2.5 μm. High-precision loss measurement was achieved using an EO controllable beam splitter to ensure the simultaneous injection of two light waves of the same input power into two waveguides of unbalanced lengths. This method avoids fluctuations in coupling efficiency when carrying conventional cut-back measurement, enabling the differentiation of two waveguides of propagation losses close to each other. Low-loss single-mode LN waveguides can be used for constructing complex photonic circuits.
